# Effects of the Ground Resolution and Thresholding on Crack Width Measurements

**DOI:** 10.3390/s18082644

**Published:** 2018-08-12

**Authors:** Hyunwoo Cho, Hyuk-Jin Yoon, Ju-Yeong Jung

**Affiliations:** 1 Robotics and Virtual Engineering, University of Science and Technology, Daejeon-si 34113, Korea; hwcho@krri.re.kr; 2 Korea Railroad Research Institute, Uiwang-si 16105, Korea; sleepii88@krri.re.kr

**Keywords:** crack width measurement, ground resolution, thresholding

## Abstract

When diagnosing the condition of a structure, it is necessary to measure the widths of any existing cracks in the structure. To ensure safety when relying on images of cracks, the selected imaging parameters and processing technology must be well understood. In this study, the effects of the ground sample distance and threshold values on the crack width measurement error are analyzed from a theoretical perspective. Here, the main source of such errors is assumed to be due to the mixed pixel phenomena in the left and right boundary pixels. Thus, a mathematical model is proposed in which the intensity changes in these pixels are computed via an equation. In addition, the relationship between the error and error probability distribution is represented with an equation based on the threshold values and mean error. Upon analysis, it was found that the threshold value that minimizes the error is at the mid-point between the background and foreground, and the probabilistic nature of the error indicates that it is theoretically possible to predict both the error and its probability distribution. The proposed model was validated using artificial images.

## 1. Introduction

The line width measurement technology that is employed in crack width management activities is commonly used in a variety of fields. For example, in the field of medicine, it is used to measure the thicknesses of blood vessels and joints, and is employed in computed tomography (CT) and magnetic resonance imaging (MRI) images [1,2,3,4,5,6,7,8,9]. In the field of machinery, it is used to measure the thicknesses of plates, oil films, cables, etc. [5,10,11]. In the field of remote sensing, it is used to measure the widths of roads and rivers [12,13], and is also used when measuring the thicknesses of pearls and analyzing nano-scale standard structures [14,15]. Crack thickness is an important criterion for evaluating the safety of structures. Factors that affect line thickness measurements include the imaging parameters, such as the resolution, and the image processing techniques, such as thresholding and edge detection.

Studies in the literature on the resolution and measurement errors in crack measurement include the following. Jahanshahi and Li et al. found that increasing the imaging distance reduces the spatial resolution of the images, and reducing the number of pixels used to represent cracks lowers the accuracy of crack measurement [16,17,18]. Jahanshahi et al. analyzed printed crack images with crack widths of 0.4–2.0 mm while varying the imaging distance from 725 to 1760 mm and found that the measurement accuracy was reduced as the number of pixels used to represent the cracks decreased. They also found that a decrease in the imaging distance, an increase in the focal distance, and an increase in the resolution are all necessary to increase the accuracy of crack measurement [17]. Li et al. proposed a technique to measure the widths of cracks in bridges from a long distance away [18], and found that as the measurement distance increased, the crack measurement error became larger, and the light source and film speed (ISO) had no effect on the magnitude of the error. 

Studies have also been conducted on the relationship between the resolution and measurement error outside of cracks. Merkx et al. analyzed the relationship between the resolution and the error when measuring the diameter of blood vessels [19]. In their study, a full width at half maximum (FWHM)-based method was used to measure the diameter of blood vessels, and they found that the resolution should be greater than 2 pixels/diameter to ensure the measurement error was less than 10%. Prevrhal et al. used an optimized segmentation method to determine how thickness measurements depend upon the spatial resolution, and found that the cortical thickness must be larger than the FWHM of the point spread function of the scanner to obtain accurate measurements [20]. Hsieh et al. analyzed the effect of the spatial resolution on the classification error [21]. In that study, the statistical features of mixed pixels were derived and the corresponding classification errors were evaluated, all of which demonstrated that a reduction in the ratio of the field width to ground sample distance generally reduced the number of classification errors. 

In addition to the resolution, threshold values are also important factors in accurate line width measurement. Treece et al. found that simple thresholding and 50% relative thresholding techniques could not produce accurate estimations in cortical bone thickness using CT data, so they proposed a new thickness estimation method [22]. Nyyssonen reported that setting improper thresholds caused errors in line width measurements, and found that setting the maximum intensity of the threshold to 25% resulted in accurate line width measurements [23]. 

In our research, we identified the following deficiencies in existing studies: existing studies on resolution and line width measurement do not consider image processing techniques for automatically extracting line widths, studies on thresholding and line width measurement do not consider the digital sampling process, and studies on resolution do not consider the influence of image processing. Thus, researchers in the previous studies were unable to show that the error is probabilistic. In addition, image-based automatic line width measurement techniques combine the sampling and extraction processes into a single step, so their combined effect on line width measurement must be considered.

In this study, the effects of the ground sample distance (GSD), which means that the actual distance per pixel, i.e., unit-length/pixel, and thresholding during image processing have on crack width measurements are analyzed from a theoretical perspective. Here, as the crack width measurement is assumed to be affected by the mixed pixel phenomena in the left and right boundary pixels, the intensity changes in the left and right boundary pixels were modeled mathematically. The intensities of the left and right boundary pixels were assumed to occur probabilistically based on the position where the pixel was measured, and the measurement errors due to the ground resolution and threshold value are evaluated in terms of the probability distribution of the error. In addition, a threshold value that was able to minimize the mean error was identified. 

## 2. Line Width Measurement Error

The width of a crack is measured as the distance from the edge of one side of a crack to the opposite edge in a direction that is perpendicular to the first edge. In digital images, the number of pixels between the edges is used to measure the width in pixel units, and the number of detected units is multiplied by the ground sample distance (GSD) to convert the measurement to actual units. This can be represented as follows:

(1) $$w_{m}=g\times N,$$

where w_m_ is the measurement value and g is the GSD in unit-length/pixel. The error in width measurements is the difference between the true and measured widths, and can be expressed as follows:

(2) $$e=w-w_{m},$$

where e is the measurement error and w is the true width. In other words, the measurement error is determined by the GSD g and the detected number of pixels N. Here, the detected number of pixels N is related to the image processing technology, and when the pixels are detected via thresholding, the detected number of pixels (N) varies depending on the thresholding value. 

To detect cracks, a suitable threshold value must be set. When crack images are measured in ideal circumstances where they are not discontinuous, the pixels representing the crack can be extracted by choosing an appropriate threshold value between the intensity of the crack and the background. However, images must be digitized for automated crack measurement, which raises the incidence of the mixed pixel phenomenon. The mixed pixel phenomenon refers to situations in which a single pixel is meant to represent one or more different intensities, but only displays a mixture of intensities. This phenomenon often occurs at boundaries. The intensities of the pixels at the boundaries of backgrounds and foregrounds are determined by the ratio of the background and foreground intensities included in the pixel as follows:

(3) $$C=A\times A_{a}+B\times B_{a},$$

where A and B are the foreground and background intensities, respectively, and A_a_ and B_a_ are the ratios of A and B included in pixel C.

**Figure 1:**
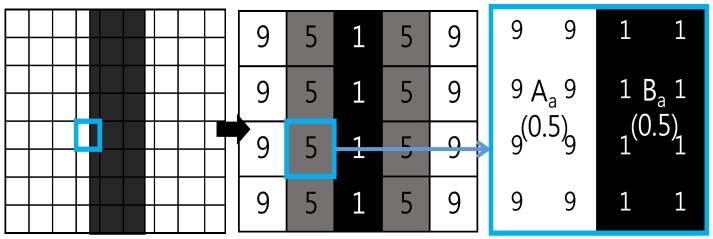
The digital image sampling process and mixed pixel phenomenon.

The mixed pixel phenomenon that occurs during the digital image sampling process is shown in Figure 1. The blue box in the figure indicates a boundary pixel where the mixed pixel phenomenon occurs. In the example shown in the figure, the background intensity is 9 and the foreground intensity is 1, and the ratio of background and foreground intensities included in the boundary pixel is 0.5. Thus, the boundary pixel intensity C can be calculated as follows: 5=1×0.5+9×0.5. As a result, the pixel located at the boundary has a value that does not reflect the intensity of the crack, but rather a value between the intensities of the background and the crack. As this phenomenon makes it difficult to detect boundary pixels when extracting cracks from images, it has a corresponding impact on the crack width measurement error.

**Figure 2:**
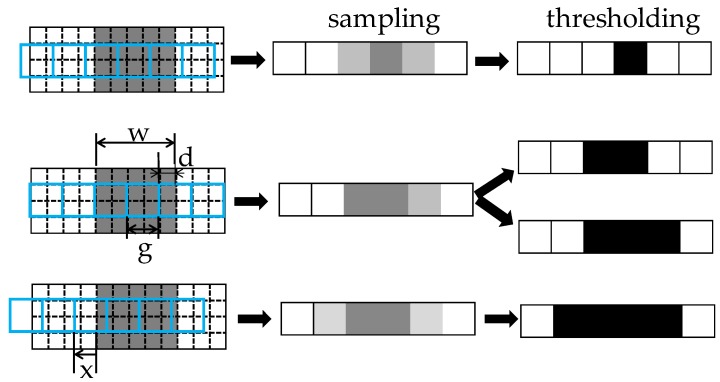
The intensity changes and pixel extraction via thresholding according to the pixel sampling position in the sample process.

The thresholding process after sampling an image is shown in Figure 2. As shown in the figure, the intensities of the pixels located at the boundary of the crack varies depending on the ratio of the background and foreground intensities included in the boundary pixels. This ratio varies depending upon the position of the pixels used to sample the image. The intensity of the boundary pixels varies according to the position of the pixels used to measure the crack, and the number of pixels that contain the foreground intensity also varies. Here, the positions of the pixels used to measure the crack are a random factor that cannot be controlled by the user. As the intensities of the boundary pixels vary, so does the number of pixels extracted via thresholding. 

**Figure 3:**
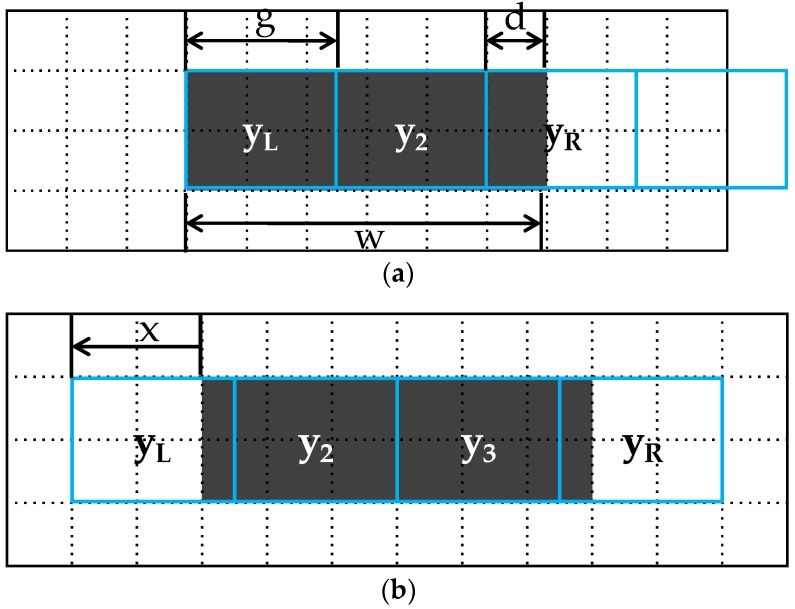
The changes in the ratio of boundary pixels which contain cracks (foreground) according to pixel sampling position: (**a**) no background intensity is included in pixel y_L_; (**b**) the pixel is moved an amount x to the left.

The process of sampling crack images is shown in Figure 3. The set of pixels used to sample the image are shown in array y_i_, where i is the index of the array. When the left boundary pixel is y_L_ and the right boundary pixel is y_R_, the ratio of the foreground and background intensities included in the boundary pixels of both sides vary depending upon the position of the sampling pixels. In Figure 3a, it can be seen that no background intensity is included in pixel y_L_; however, when the pixel is moved an amount x to the left, as in Figure 3b, the proportion of background intensity included increases to more than the foreground intensity. Not only does the ratio of the included pixels change, the number of pixels that include the foreground intensity (i.e., the crack) also changes. If the index L of the left boundary pixel is assumed to be one, the index of the right boundary pixel R in Figure 3a is three. Here, if the position of the sampling pixel is moved x amount to the left, as in Figure 3b, the index R of the right boundary pixel increases to four. Figure 3 is shown to help understand Equations (4)–(10), and the symbols in all the figures presented in this paper are the same as those used in the equations.

The following equations show the changes in intensity due to the mixed pixel phenomena in the left and right boundary pixels as they change according to the position of the sampling pixels as per Figure 3.

(4) α=(A−B),

(5) $$q=\frac{w}{g},$$

(6) $$\mathrm{if}\;0\leq x<1:\;y_{L}\left(x\right)=\alpha x+B,\;L=0,$$

(7) $$\mathrm{if}\;0\leq x<1-d:\;y_{R}\left(x\right)=-\alpha x-\alpha d+A,\;R=\lceil q\rceil ,$$

(8) $$\mathrm{if}\;1-d\leq x<1:\;y_{R}\left(x\right)=B,\;R=\lceil q\rceil ,$$

(9) $$y_{R}\left(x\right)=-\alpha x-\alpha d+2A-B,\;R=\lceil q\rceil +1,$$

where y_L_ and y_R_ are the intensities of the left and right boundary pixels, respectively; L and R are the indexes of the left and right pixels, respectively; and x is the ratio of the background intensity A included in y_L_ or the pixel shifting ratio and describes the changes in intensity versus the sampling position. Here, d is the ratio of the foreground intensity B included in y_R_ when x = 0, and is defined as follows:

(10) $$d=\frac{\mathrm{mod}\left(w,g\right)}{g}=1+\frac{\left(w-g\lceil \frac{w}{g}\rceil \right)}{g}.$$

When the index of the left boundary pixel L is assumed to be zero, the index of the right boundary pixel R can be expressed as q, which is the crack width w divided by GSD g (Equation (5).) Equations (7) and (9) represent the conditions in which the index of the right boundary pixel increases or conditions in which the number of pixels included in the foreground intensity increases. When the index of the left boundary pixel L is assumed to be zero, the index of the right boundary pixel R is ⌈q⌉. When x becomes larger than 1 − d, R increases to ⌈q⌉+1. 

Therefore, the number of pixels that include the foreground intensity is R−L, and the number of pixels N detected via thresholding is defined according to whether the left and right boundary pixels are detected, as follows.

(11) N=R−L+k, k=−2,−1,0,

where k is the number of detected left and right boundary pixels. When all pixels on both sides are detected, k = 0, and when all pixels are not detected, k = −2. In general, L = 0, and R is in the range of (⌈q⌉, ⌈q⌉+1); thus, N is in the following range:

(12) N∈(⌈q⌉−2, ⌈q⌉+1), N∈ℤ.

Here, N has four possible values; thus, the measurement error e also has four cases:

(13) $$e_{0}=w-g\left(\lceil q\rceil -2\right)=w-g\lceil q\rceil +2g,$$

(14) $$e_{1}=w-g\left(\lceil q\rceil -1\right)=w-g\lceil q\rceil +g,$$

(15) $$e_{2}=w-g\lceil q\rceil ,$$

(16) $$e_{3}=w-g\lceil q\rceil -g.$$

Equation (10) shows that w−g⌈q⌉=g(d−1), so the above equations define the following equations for d.

(17) $$e_{0}=g\left(d+1\right),\;\mathrm{for}\;N=\lceil q\rceil -2,$$

(18) $$e_{1}=g\left(d\right),\;\mathrm{for}\;N=\lceil q\rceil -1,$$

(19) $$e_{2}=g\left(d-1\right),\;\mathrm{for}\;N=\lceil q\rceil ,$$

(20) $$e_{3}=g\left(d-2\right),\;\mathrm{for}\;N=\lceil q\rceil +1.$$

## 3. Probability Distribution of the Measurement Error 

The previous section examined cases in which an error can occur due to the threshold values. This section discusses the distribution of the probability that an error occurs. The reason that the probability of an error occurring varies is that the intensities of the left and right boundary pixels change depending on the position of the sampling pixels. Changes in the positions of the sampling pixels are represented by x in Equations (6)–(9). The range of x in which the intensities of y_L_(x) and y_R_(x) are less than T indicates the probability of the pixel being extracted. When a threshold value T is selected, the sections in which each of the boundary pixels is extracted are as follows:

(21) $$V_{1}={\{x|y}_{L}\left(x\right)<T\},$$

(22) $$V_{2}=\{x|y_{\left\lceil q\right\rceil }\left(x\right)<T\},$$

(23) $$V_{3}=\left\{{x|y}_{\lceil q\rceil +1}\left(x\right)<T\right\}.$$

The probability that each error occurs can be defined through set theory, as follows:

(24) $$P\left(e_{0}\right)=P\left({\left(V_{1}\cup V_{2}\cup V_{3}\right)}^{c}\right),$$

(25) $$P\left(e_{1}\right)=P\left(\left(V_{1}^{c}\cap V_{2}\cap V_{3}^{c}\right)\cup \left(V_{1}\cap V_{2}^{c}\cap V_{3}^{c}\right)\right),$$

(26) $$P\left(e_{2}\right)=P\left(\left(V_{1}^{c}\cap V_{2}\cap V_{3}\right)\cup \left(V_{1}\cap V_{2}\cap V_{3}^{c}\right)\right),$$

(27) $$P\left(e_{3}\right)=P\left(V_{1}\cap V_{2}\cap V_{3}\right),$$

where V_1_, V_2_, and V_3_ indicate the sections of x in which the left and right boundary pixels are extracted, respectively. More specifically, V_1_ indicates the section where the left boundary pixel is extracted, and V_2_ and V_3_ indicate the sections where the right boundary pixels with indices ⌈q⌉ and ⌈q⌉+1 are extracted. P(e) indicates the probability of error occurrence.

**Figure 4:**
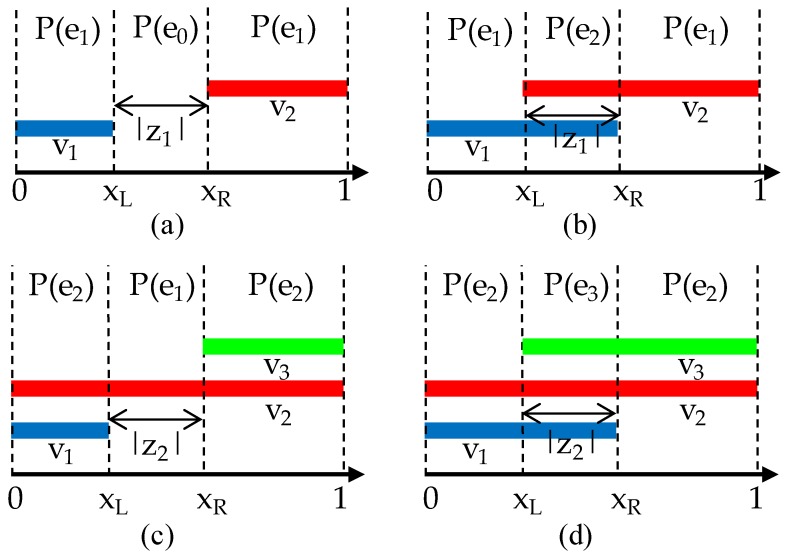
Sections of the left and right boundary pixels extracted based on the threshold value and error probability distribution: (**a**) P_1_(e); (**b**) P_2_(e); (**c**) P_3_(e); and (**d**) P_4_(e).

Figure 4 shows Equations (24)–(27) by way of a diagram, and set theory is used to describe the relationships between the errors and their probability distributions. Here, x_L_ and x_R_ are $T=y_{L}\left(x_{L}\right)$ and $T=y_{R}\left(x_{R}\right)$, and z_1_ or z_2_ represent the differences between x_L_ and x_R_. In Figure 4, the error probability distribution is divided into four types, and each type includes only two of the four errors. Additionally, the probability distributions of the two errors are represented by z_1_ or z_2_ and the remaining ratio. The error e_c_ indicated by z_1_ or z_2_ varies according to the type of probability distribution. The four types of probability distribution are represented as P_s_(e), s = 1, 2, 3, and 4, and the range of the threshold values T_s_ that correspond to each probability distribution type are as follows:

(28) $$\{\begin{array}{cc} B<T_{1}<y_{L}\left(t_{1}\right) & P_{1}\left(e\right)\\ y_{L}\left(t_{1}\right)<T_{2}<y_{R}\left(0\right) & P_{2}\left(e\right)\\ \begin{array}{c} y_{R}\left(0\right)<T_{3}<y_{R}\left(t_{1}\right)\\ y_{R}\left(t_{2}\right)<T_{4}<A \end{array} & \begin{array}{c} P_{3}\left(e\right)\\ P_{4}\left(e\right) \end{array} \end{array}.$$

**Figure 5:**
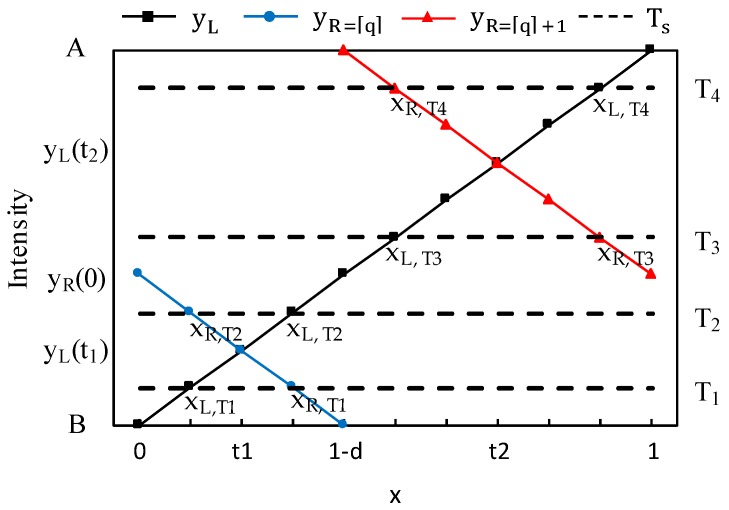
Changes in the intensities of the left and right boundary pixel as per the mixed pixel phenomenon and sampling position x.

Figure 5 shows a graph of the y_L_(x) and y_R_(x) intensity changes in Equations (6)–(9), where x_L,Ts_ and x_R,Ts_ represent $T_{s}=y_{L}\left(x_{L,\mathrm{Ts}}\right)$ and $T_{s}=y_{R}\left(x_{R,\mathrm{Ts}}\right)$, respectively. In Figure 4, it was confirmed that the probability distribution of error e_c_ had four types, and Figure 5 describes the relationships between the four types of distribution and the threshold values. In order to represent this type of probability distribution in the form of an equation, t_1_ and t_2_, which are the intersection points of y_L_ and y_R_, and x_L_, are defined as follows:

(29) $$t_{1}=-\frac{d}{2}+\frac{1}{2}=\frac{1-d}{2},$$

(30) $$t_{2}=-\frac{d}{2}+1,$$

(31) $$x_{L}=\frac{T-B}{A-B}.$$

Lines y_L_ and y_R_ are symmetrical with respect to the intersection point at the center, and z_1_ and z_2_, which are important distances for finding the error probability distribution, can be found as follows:

(32) $$z_{1}=2\left(t_{1}-x_{L}\right),$$

(33) $$z_{2}=2\left(t_{2}-x_{L}\right).$$

The case where the probability distribution P_s_(e) occurs is redefined from the intensity range in Equation (28) to the range of x, and this probability distribution is shown as the following equations:

(34) $$\mathrm{If}\;0\leq x_{L}<t_{1},\;\{\begin{array}{c} P\left(e_{0}\right)=z_{1}\;\;\;\\ P\left(e_{1}\right)=1-z_{1}\\ P\left(e_{2}\right)=0\;\;\;\;\\ P\left(e_{3}\right)=0\;\;\;\; \end{array},$$

(35) $$\mathrm{If}\;t_{1}\leq x_{L}<1-d,\;\{\begin{array}{c} P\left(e_{0}\right)=0\;\;\;\;\\ P\left(e_{1}\right)=1+z_{1}\\ P\left(e_{2}\right)=-z_{1}\;\;\\ P\left(e_{3}\right)=0\;\;\;\; \end{array},$$

(36) $$\mathrm{If}\;1-d\leq x_{L}<t_{2},\;\{\begin{array}{c} P\left(e_{0}\right)=0\;\;\;\;\\ P\left(e_{1}\right)=z_{2}\;\;\;\\ P\left(e_{2}\right)=1-z_{2}\\ P\left(e_{3}\right)=0\;\;\;\; \end{array},$$

(37) $$\mathrm{If}\;t_{2}\leq x_{L}<1,\;\{\begin{array}{c} P\left(e_{0}\right)=0\;\;\;\;\\ P\left(e_{1}\right)=0\;\;\;\\ P\left(e_{2}\right)=1+z_{2}\\ P\left(e_{3}\right)=-z_{2}\;\; \end{array}.$$

It is possible to use the equations for the error and probability distribution to express the measurement values more accurately in the form of $w_{m}\pm e_{c}\left(P_{s}\left(e_{c}\right)\right)$. Furthermore, the proposed measurement error Equations (17)–(20) are defined as functions of GSD(g) and crack width (w). Thus, the effect of GSD error on the crack measurement error can also be estimated from the proposed theory.

## 4. Mean Measurement Error According to Threshold Value

The previous section examined the error and error probability distribution according to the threshold values and ground resolution. This section discusses the threshold value T in which the error and probability distribution are employed to determine the smallest mean error. The mean error can be calculated as the product of the error and its probability distribution, as shown below.

(38) $$E\left(e\right)=\overset{3}{\underset{c=0}{\sum }}P_{s}\left(e_{c}\right)\times e_{c}.$$

The error and its probability distribution can be expressed as the function f of the background and foreground intensities A and B, the threshold value T, and d, as shown below.

(39) e, P(e)=f(A, B, T, d),

where d is a function of the true width(w) and the GSD(g) (Equation (10)). Here, w is the unknown value that is to be measured. If w is assumed to be distributed in a fixed range, the d can be assumed to be in the range of 0 to 1. Therefore, to find the threshold value T that can minimize the mean measurement error, the mean error E(e) is integrated over the interval d ∈ (0, 1) and the absolute value is taken. The threshold that can minimize this value is referred to as T^*^, which can be described as follows:

(40) $$T^{*}=argmin\;\left|\int_{0}^{1}E\left(e\right)dd\right|,$$

where P_s_(e) is divided into four types. The integral interval according to Ps is derived from the ranges in Equations (34)–(37), and E(e) is divided by x_L_ = 0.5 to simplify the calculations.

(41) $$\frac{1}{g}\int_{0}^{1}E\left(e\right)dd=\{\begin{array}{ll} \int_{0}^{1-2x_{L}}P_{1}\left(e\right)e\;dd+\int_{1-2x_{L}}^{1-x_{L}}P_{2}\left(e\right)e\;dd+\int_{1-x_{L}}^{1}P_{3}\left(e\right)e\;dd, & \mathrm{for}\;x_{L}\leq 0.5\\ \int_{0}^{1-x_{L}}P_{2}\left(e\right)e\;dd+\int_{1-x_{L}}^{2-2x_{L}}P_{3}\left(e\right)e\;dd+\int_{2-2x_{L}}^{1}P_{4}\left(e\right)e\;dd, & \mathrm{for}\;x_{L}\geq 0.5 \end{array}$$

(42) $$\left|\int^{}E\left(e\right)\right|=\left|g\left(-2.0x_{L}+1.0\right)\right|,$$

where the absolute integral mean error is described as a function of g and x_L_, and the value of x_L_ that minimizes the absolute integral error is 0.5. Therefore, the threshold value T that minimizes the mean error E(e) can be found using Equation (31).

(43) $$T^{*}=\frac{A+B}{2}.$$

From Equation (43), it can be seen that the threshold value minimizing the error is defined by the intensity of the foreground and background. 

**Figure 6:**
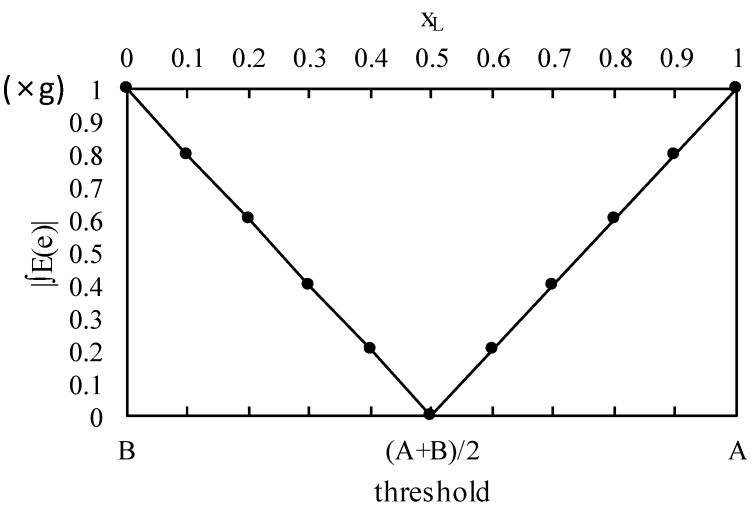
The threshold value vs. absolute integral mean error.

Figure 6 shows the relationship between the threshold value and the mean error, and it also shows that a minimum value exists. In the results, it can be seen that the mid-point between background A and foreground B intensities is the threshold value T^*^ that minimizes the mean error, and as the threshold value approaches the background or foreground intensities, the mean error increases. Additionally, when only the ground resolution is considered, the line width measurement error is between the GSD g and zero. 

## 5. Results and Discussion

**Figure 7:**
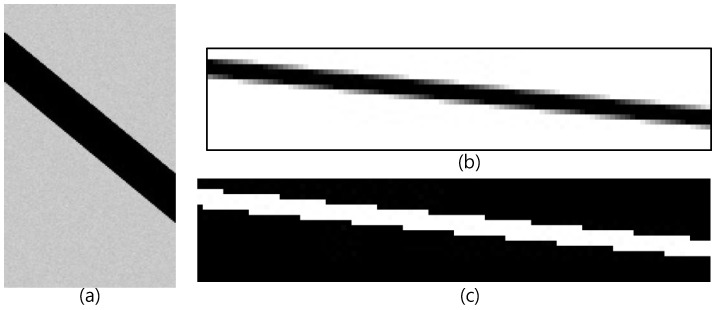
The artificial images used for crack width measurement verification: (**a**) original image, (**b**) 1/10 downsampled image, and (**c**) the results of the thresholding image.

An artificial image was used to verify the error and the probability distribution of the error caused by the derived threshold value. The sampling process employed on the artificial image simulated a process of creating a high resolution original image and then downsampling it to digitize the image. The original image is shown in Figure 7a. The intensity of the background in the original image was set at a mean of 200 with a standard deviation of 1, and the intensity of the crack was set at 0. The width of the crack was set at 35 pixels. The previously presented equation has no limitations to the input values, including intensity, width, and GSD, except for the condition where the intensity of the crack is lower than that of the background. Therefore, the intensity, width, and GSD of the sample image were randomly determined and do not have any special meaning. In the original image, there is a diagonal line of pixels extending from the upper left to the lower right. This made it possible to observe the intensity changes in the boundary pixels based on the positions of the sampling pixels. 

The original image was downsampled at a ratio of 1/10 to simulate sampling at a ground resolution where the GSD was 10 pixels/pixel. The result is shown in Figure 7b. Note that the downsampling was only performed on the rows. In the figure, it can be seen that the intensities of the boundary pixels on either side increased or decreased slightly. A thresholded image of the sampled image in Figure 7b is shown in Figure 7c, in which the identified crack is shown in white. The theoretical values of the measurement error in the crack width (w) and the probability distribution of the error based on the threshold value T are shown in Table 1, in which the measured crack width w_m_ is shown as w_m_ + e(P(e)). 

The measurement error of the crack width and the probability distribution of the error when the simulated image was thresholded are shown in Table 2. The standard deviation of the background intensity was computed to determine the probability distribution of the error that occurred in five measurements. 

**Figure 8:**
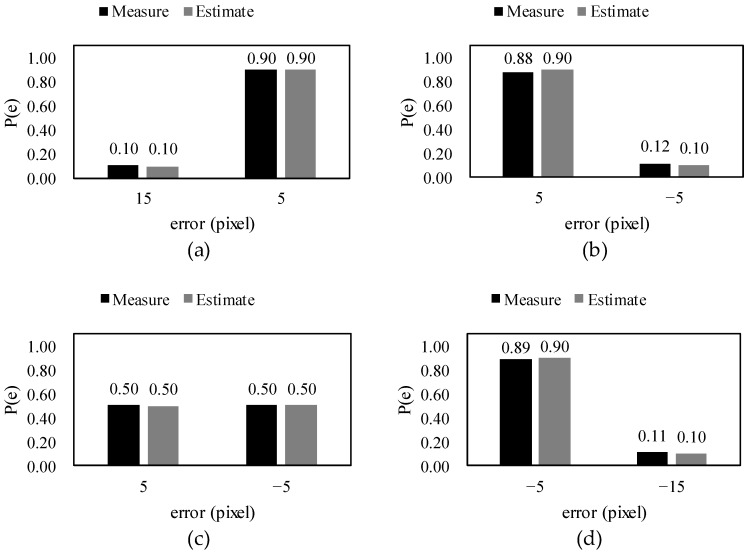
The theoretically estimated values and simulation measurement values of crack width measurement error and error probability distribution according to threshold value: (**a**) threshold value of T = 40; (**b**) threshold value of T = 60; (**c**) threshold value of T = 100; and (**d**) threshold value of T = 160.

Graphs of the theoretically estimated and measured values of the error and associated probability distribution are shown in Figure 8, where it can be seen that the actual measured values were almost the same as the theoretical values. In addition, the difference between the measured and estimated values at other GSDs are given in Appendix A. Figure 8a–d correspond to probability distribution types P_1_, P_2_, P_3_, and P_4_, respectively, and the probability distribution type according to the threshold value can be found via Equation (28). The process of calculating the error and its probability distribution is as follows. 

Before the error is found via Equations (17)–(20), d is found using Equation (10) as follows:

d = mod(35, 10)/10 = 0.5.

The crack width measurement error can be calculated using Equations (17)–(20) as follows:

e_0_ = 10(0.5 + 1) = 15,

e_1_ = 10(0.5) = 5,

e_2_ = 10(0.5 − 1) = −5,

e_3_ = 10(0.5 − 2) = −15.

When the width is 35 pixels and the GSD is 10 pixels, the maximum error is ±15 pixels and the minimum error is ±5 pixels. In the case of the minimum error, the GSD is not an integer multiple of the width, so an error with a magnitude of d is unavoidable as this is the remainder when dividing the width by the GSD. The maximum error occurs in conditions when all of the left and right boundary pixels are either extracted or not extracted. For example, if none of the pixels from the two boundaries are extracted, a 2 × 10, or 20, pixel error occurs. This is reduced by d to a 15 pixel error. 

The probability distribution of the error can be found using Equations (34)–(37). To find the probability distribution of the error, t_1_, t_2_, and x_L_ must first be calculated using Equations (29)–(31). Since t_1_ and t_2_ are functions of d, they can be found from the crack width and GSD, as follows:

t_1_ = (1 − 0.5)/2 = 0.25,

t_2_ = −0.5/2 + 1 = 0.75.

The value of x_L_ varies depending on the threshold value, and determines the probability distribution of the error. Examples of how the error occurs depending on the threshold value probability distribution of the error are as follows.

When the threshold value is T = 40,

x_L_ = (40 − 0)/(200 − 0) = 0.2,

which satisfies the conditions of Equation (34), and the probability distribution of the corresponding error is:

P(e_0_) = 2(0.25 − 0.2) = 0.10,

P(e_1_) = 1 − 0.1 = 0.9.

When the threshold value is T = 60,

x_L_ = (60 − 0)/(200 − 0) = 0.3,

which satisfies the conditions of Equation (35), and the probability distribution of the corresponding error is:

P(e_1_) = 1 + 2(0.25 − 0.3) = 0.9,

P(e_2_) = −2(0.25 − 0.3) = 0.1.

When the threshold value is T = 100,

x_L_ = (100 − 0)/(200 − 0) = 0.5,

Which satisfies the conditions of Equation (36), and the probability distribution of the corresponding error is:

P(e_1_) = 2(0.75 − 0.5) = 0.5,

P(e_2_) = 1 − 0.5 = 0.5.

When the threshold value is T = 160,

x_L_ = (160 − 0)/(200 − 0) = 0.8,

which satisfies the conditions of Equation (37), and the probability distribution of the corresponding error is,

P(e_2_) = 1 + 2(0.75 − 0.8) = 0.9,

P(e_3_) = −2(0.75 − 0.8) = 1.

As shown in the examples above, if the crack width to be measured, the GSD and the background and foreground intensities are known, the error in the width measurement and the probability distribution of that error occurring can be calculated. 

**Figure 9:**
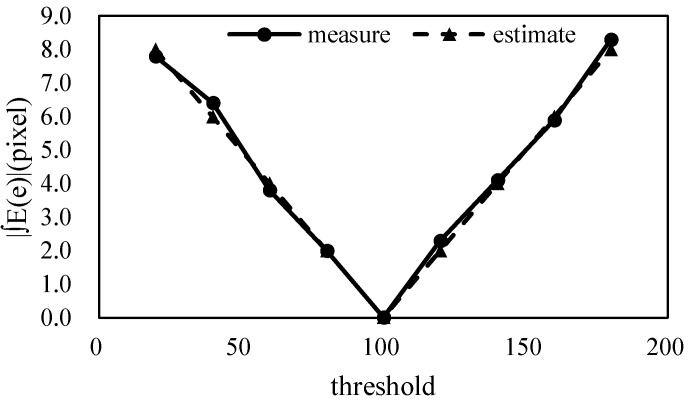
The measured and theoretically estimated values of the mean error E(e) based on the threshold value.

Figure 9 shows the measured and estimated values using Equation (42) to compute the mean error according to the threshold value. In the figure, it can be seen that the measured and estimated values were almost the same. It can also be seen that the mid-point between the foreground and background values is the threshold value at which the error is minimized, as is predicted by Equation (43).

**Figure 10:**
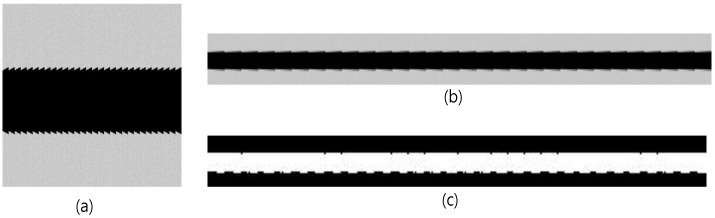
The virtual images for verifying mean crack width measurement: (**a**) original image; (**b**) 1/10 downsampled image; and (**c**) results of thresholding image.

Equation (42) for the mean crack width is also valid in situations where the width is variable. The actual crack width and other lines do not appear at an even width anywhere in the entire section. In such cases, the variable width is averaged to find the mean width. Figure 10 shows a virtual image for confirming that the relationship equation for the mean error according to the threshold value is valid when the mean width of a variable-width crack is found. Figure 10a was created with the width evenly distributed from 100 to 110, and the mean width was 105. The background intensity had a mean of 200 and a standard deviation of 1, and the foreground intensity was 0. 

Once created, the original image in Figure 10a was downsampled by 1/10 to simulate the image digitization process. The corresponding downsampled image is shown in Figure 10b. Figure 10c shows a thresholded image of the sampled image.

**Figure 11:**
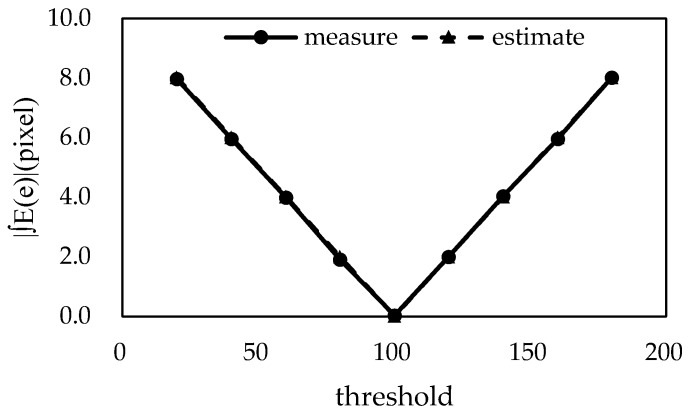
The mean error (E(e)) of the variable crack width according to threshold value in measured values and theoretically estimated values.

Figure 11 shows the mean width measurement results for the threshold values of the virtual image with a variable width. It can be seen that the theoretically estimated and measured values were almost the same, and the error became smaller at the median value between the intensity of the background and foreground (crack). 

Additionally, an experiment using real images was performed to verify the proposed theory. In the experiment, printed cracks were drawn and printed with the desired width for precise crack width control. The printed crack, with a width of 3 mm, was measured using a scanner (iR ADV C5540i, Canon Inc, Tokyo, Japan) with a resolution of 600 dpi. The scanner was used to minimize external influences. 

**Figure 12:**
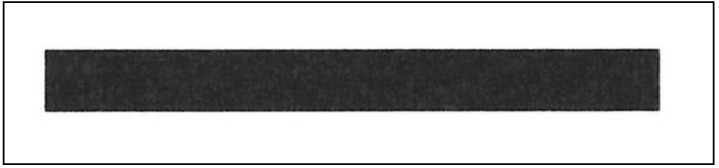
Printed crack image measured with digital scanner.

Figure 12 shows a printed crack image measured using the scanner. The intensity of the background and the foreground in the measured image were 245 and 45, respectively. The measured image is affected by the blurring of the lens during the sampling process. To reduce the effects of uncontrollable elements, the measured images were downsampled to 1/10 and analyzed. The results of the theoretical estimates and the measurements are listed in Table 3. From the table, it can be seen that the measured values were almost the same as the theoretical values.

## 6. Conclusions

In this study, the effects of the ground resolution and thresholding values on crack width measurement errors are analyzed from a theoretical perspective. An equation was derived to show the changes in the intensities in the left and right boundary pixels due to the mixed pixel phenomenon, an equation describing the probability distribution of the error was derived, and an equation describing the relationship between the threshold value and mean error was derived. The results showed that the threshold value that minimizes the error is the mid-point between the foreground and background intensities, and the mean measurement error was seen to increase as the threshold value approached the background or foreground intensity values. 

The proposed theory was validated using artificial images. A virtual line with a set width and intensity was defined, and was then downsampled to simulate the image digitization process. The crack width in the simulated image was then measured, and a comparison was made with the results of the proposed equations, which indicated the theoretical and measured values were almost the same. 

In the case of continuous signals, the cracks are measured in a standard way regardless of the threshold value; however, in the case of digital sampling, it was demonstrated that the measured values are distributed probabilistically based on the threshold value. It was also proven that it is possible to mathematically predict this probability distribution, and when the crack width is repeatedly measured and an equation for producing the mean is used, it is theoretically possible to ensure the measurement error is equal to zero.

This study only dealt with the ground resolution and threshold for the causes of line width measurement errors from using digital images. In order to accurately predict the actual crack width measurement errors, the effects of factors such as edge roughness and lens blurring on the intensity of boundary pixels must be considered as well. However, it is very difficult to mathematically model these phenomena. Furthermore, the proposed equations were derived by assuming one dimension. However, two-dimensional factors such as the measured crack angle and shape can affect the prediction of measurement errors. 

Therefore, the proposed theory could be used to estimate the theoretical limits of the measurement errors by using the approximated information of the cracks to be measured. In future, it is expected that more accurate error prediction is available if the effects of factors such as lens blurring and two-dimensional shapes are considered. 

## Figures and Tables

**Table 1:** The theoretical estimations of measurement values, error and error probability distributions due to threshold values.

Threshold Value	Measurement Value (w_m_)	Error Type (e_i_)
40	20 + 15(0.1)	e_0_
	30 + 5(0.9)	e_1_
60	30 + 5(0.9)	e_1_
	40 − 5(0.1)	e_2_
100	30 + 5(0.5)	e_1_
	40 − 5(0.5)	e_2_
160	40 − 5(0.9)	e_2_
	50 − 15(0.1)	e_3_

**Table 2:** The error type and probability distribution based on the threshold value of the virtual image.

Threshold Value	P(e)						Error Type (e_i_)
	1	2	3	4	5	Mean	
40	0.11	0.12	0.08	0.8	0.12	0.10	e_0_
	0.89	0.88	0.92	0.92	0.88	0.90	e_1_
60	0.9	0.86	0.89	0.91	0.85	0.88	e_1_
	0.1	0.14	0.11	0.09	0.15	0.12	e_2_
100	0.5	0.5	0.49	0.53	0.49	0.50	e_1_
	0.5	0.5	0.51	0.47	0.51	0.50	e_2_
160	0.9	0.87	0.89	0.9	0.89	0.89	e_2_
	0.1	0.13	0.11	0.1	0.11	0.11	e_3_

**Table 3:** The theoretically estimated values and measured values of error probability distribution using real image (ΔP(e) = Estimation − Measurement).

Threshold	Error Type	P(e)		∆P(e)
		Estimation	Measurement	
100	e_0_	0.35	0.37	−0.02
	e_1_	0.65	0.63	0.02
180	e_1_	0.55	0.6	−0.05
	e_2_	0.45	0.4	0.05

## Appendix A

**Table A1:** The theoretically estimated values and measured values of error probability distribution depending on GSD(ΔP(e) = Estimation − Measurement).

GSD (Pixels/Pixel)	Threshold	Error Type	P(e)		∆P(e)
			Estimation	Measurement	
5	40	e_0_	0.6	0.63	−0.03
		e_1_	0.4	0.37	0.03
	60	e_0_	0.4	0.4	0
		e_1_	0.6	0.6	0
	100	e_1_	1	1	0
	160	e_1_	0.4	0.402	−0.002
		e_2_	0.6	0.598	0.002
20	40	e_0_	0.85	0.848	0.002
		e_1_	0.15	0.152	−0.002
	60	e_1_	0.65	0.648	0.002
		e_2_	0.35	0.352	−0.002
	100	e_1_	0.25	0.246	0.004
		e_2_	0.75	0.754	−0.004
	160	e_2_	0.65	0.662	−0.012
		e_3_	0.35	0.338	0.012

Table A1 lists the theoretically estimated values and measured values of error probability distribution depending on the GSD. It can be seen that the estimated and measured values are similar, regardless of the GSD. Here, a GSD 5 and 20 were implemented by down-sampling the images in Figure 7 to 1/5 and 1/20, respectively.
